# Interoception-Based Interventions for Executive Functioning and Behavioral Outcomes in Children and Young Adults: A Scoping Review

**DOI:** 10.3390/pediatric18050122

**Published:** 2026-09-22

**Authors:** Christina Pliatsika, Olympia Vasileiadou, Konstantina Avgerinou, Niki Lazi, Pantelis Pergantis, Konstantinos Georgiou, Georgios Kalogirou, Andreas Petropoulos, Georgios Tsigaras, Athanasios Drigas

**Affiliations:** 1 Faculty of Medicine, School of Health Sciences, University of Thessaly, 41334 Larissa, Greece; konstantina.avg@outlook.com; 2 Faculty of Social Sciences, Hellenic Open University, 26335 Patras, Greece; vasileiadoyolympia@gmail.com; 3 Occupational Therapy Department, School of Health Sciences, University of Western Macedonia, 50200 Ptolemaida, Greece; laziniki12@gmail.com (N.L.); kgeorgiou@uowm.gr (K.G.); gio.kalogirou86@gmail.com (G.K.); 4 Net Media Lab & Mind & Brain R&D, Institute of Informatics & Telecommunications, National Centre of Scientific Research ‘Demokritos’, 15341 Athens, Greece; dr@iit.demokritos.gr; 5 Department of Physiotherapy, School of Health Sciences, University of Thessaly, 35132 Lamia, Greece; petropoulos_andreas@yahoo.gr; 6 Department of Physiotherapy, School of Health Sciences, International Hellenic University, 57400 Thessaloniki, Greece; tsigarasphysiopediatric@gmail.com

**Keywords:** interoception, executive functioning, self-regulation, behavioural outcomes, occupational therapy

## Abstract

**Background/Objectives:** Interoception-based interventions aim to help individuals recognize and respond to internal bodily signals. Their contribution to executive functioning and behavioral regulation remains unclear. This scoping review mapped interventions and reported outcomes in children, adolescents, and young adults. **Methods:** PubMed, Scopus, and Web of Science were searched for English-language studies published between 2015 and 3 May 2026. The review followed Joanna Briggs Institute guidance and PRISMA-ScR reporting recommendations. Two reviewers independently screened records and extracted data. Findings were synthesized narratively; no formal critical appraisal was undertaken. **Results:** Nine studies included participants aged 4–23 years across clinical and nonclinical populations. Approaches included structured interoception curricula, acceptance-based exposure, and programs combining bodily awareness with other therapeutic components. Three studies assessed executive functioning using the Behavior Rating Inventory of Executive Function–Second Edition. Reported gains mainly concerned emotional and behavioral regulation, although improvements in some rated cognitive domains were also described. These findings did not establish improvements on performance-based executive function tasks. Other reported changes concerned anxiety, pain, eating, and social functioning. Findings were not uniformly positive: physiological outcomes were mixed, and one study reported increased worry about bodily sensations. Small samples, limited controlled comparisons, heterogeneous measures, and scarce follow-up data restricted interpretation. **Conclusions:** The literature reports preliminary improvements in regulation and selected behavioral outcomes but does not establish intervention effectiveness or the specific contribution of interoception. Controlled studies should assess both everyday regulation and executive task performance, isolate intervention components, and examine whether benefits persist.

## 1. Introduction

Interoception constitutes a fundamental sensory system through which the brain receives information about the physiological state of the body, contributing to the maintenance of homeostasis and adaptive behaviour [1,2]. Recent evidence recognises interoception as an important mechanism associated with emotion regulation, as it refers to the ability to perceive and interpret internal physiological signals of the body, such as heart rate, respiration, fatigue, and body temperature [2,3,4]. The accurate interpretation of these signals appears to contribute significantly to more effective self-regulation, in contrast to the adjustment difficulties that may arise from reduced interoception [4,5].

Beginning in childhood and extending into early adulthood [6], the development of self-regulation and emotion regulation skills is crucial, as these influence learning, social relationships and interactions, and overall psychosocial development [7,8], especially in a modern world characterised by rapidly changing demands. Effective regulation requires the recognition and understanding of one’s emotional state in order to apply strategies that modify emotional responses into more appropriate ones whenever necessary [3]. Difficulties in self-regulation are directly associated with increased levels of anxiety [5,9,10], executive functioning deficits, behavioural problems and reduced performance in functional activities [3,7,8,10]. Effective self-regulation is closely associated with the proper functioning of executive functions, which play a key role in cognitive and behavioural control [11]. Executive functions are defined as the set of cognitive processes that support goal-directed behaviour [6,12,13,14] and are associated with the functioning of the prefrontal cortex [6,15], including the regulation of thoughts and actions [13].

Furthermore, recent systematic reviews have shown that interoceptive impairments are associated with a broad range of mental health difficulties [3,16,17] and neurodevelopmental disorders during childhood and adolescence [7,17]. Interoception-based interventions are founded on the premise that enhancing interoception will lead to positive emotional and behavioural outcomes, particularly in individuals who are still developing during childhood and early adulthood [2,4,18,19].

The existing literature is limited in terms of both primary studies and reviews, making it difficult to draw critical comparisons across findings. Furthermore, the available studies implement heterogeneous interventions targeting multiple domains, as interoception can be involved in various aspects of human functioning, including emotional processes, biological functions, and other physiological mechanisms.

The aim of this scoping review was to explore the current evidence on interoception-based interventions in children and young adults by identifying the interventions implemented and examining their impact on executive functioning and behavioural outcomes. The following research questions were formulated:

(RQ1) What types of interoception-based interventions have been implemented in children and young adults?

(RQ2) What effects do interoception-based interventions have on executive functioning in children and young adults?

(RQ3) What effects do interoception-based interventions have on behavioural outcomes in children and young adults?

Based on the existing literature, it was expected that interoception-based interventions would demonstrate beneficial effects on executive functioning and behavioural outcomes in children and young adults, while the formulations of the programmes would differ from one another. It was also hypothesised that considerable heterogeneity would be observed across studies in terms of intervention type, target populations, and outcome measures. Previous reviews suggest that interoception-based interventions predominantly focus on emotional outcomes, while executive functioning has received comparatively limited attention in both intervention design and outcome evaluation.

## 2. Materials and Methods

### 2.1. The Study Design

A scoping review was conducted to map and synthesize the available evidence on interoception-based interventions and their impact on executive functioning and behavioral outcomes in children and young adults. The review followed the methodological framework of [20], with enhancements proposed by Levac et al. [21] and guidance from the Joanna Briggs Institute [22]. Reporting adhered to the PRISMA-ScR guidelines [23]. The methodology was selected due to its suitability for mapping emerging and heterogeneous evidence and identifying gaps in the literature [22,24]. The review protocol was registered on the Open Science Framework (OSF) (DOI: 10.17605/OSF.IO/AHWPB).

### 2.2. Eligibility Criteria

Studies were eligible for inclusion if they: (1) included children, adolescents, or young adults with typical development or clinical conditions; (2) examined an interoception-based intervention, either as a stand-alone intervention or as part of a broader therapeutic programme; (3) reported outcomes related to executive functioning and/or behavioural outcomes; and (4) employed quantitative, qualitative, or mixed-methods primary research designs, including RCTs, quasi-experimental studies, pilot and feasibility studies, pre/post-intervention studies, and case reports.

Studies were excluded if they: (1) investigated interoception or interoceptive awareness without implementing an intervention; (2) evaluated interventions that were not primarily based on interoception; (3) focused exclusively on adults; (4) were review articles, study protocols, conference abstracts, editorials, dissertations, or opinion papers; or (5) were not published in English.

### 2.3. Information Sources and Search Strategy

A comprehensive literature search was conducted to identify studies relevant to the research question. PubMed, Scopus, and WoS served as the primary databases. The literature search was conducted on 3 May 2026. The search strategy combined controlled vocabulary (where applicable) and free-text keywords related to interoception, children and adolescents, interventions, executive functioning, and behavioural outcomes, using the Boolean operators AND and OR. Searches were limited to studies published between 2015 and 3 May 2026 and articles available in the English language. The complete database-specific search strategies, including all search terms, Boolean operators, and database-specific field codes, are reported in Appendix A (Table A1) to support reproducibility of the search.

### 2.4. Study Selection Process

The records identified through the database searches were imported into Rayyan [25], where duplicate records were removed. Title and abstract screening was conducted independently by two reviewers according to the predefined eligibility criteria. Potentially eligible studies were subsequently assessed in full text. Disagreements between reviewers were resolved through discussion and consensus or, when necessary, through consultation with a third reviewer.

### 2.5. Data Extraction

Data extraction was conducted independently by two researchers using a standardized data extraction form developed for this review. Any discrepancies between reviewers were resolved through discussion and consensus, or, when necessary, by consultation with a third reviewer.

The following data were extracted from each included study: author(s) and year of publication, study aim, study design, population, sample size (N), age, intervention provider, intervention, duration and frequency of the intervention, assessment tools, executive functioning outcome measures, behavioural outcome measures, executive functioning findings, and behavioural findings.

### 2.6. Data Synthesis

A narrative synthesis approach was used to summarise and present the findings of the included studies. Due to the heterogeneity of study designs, populations, interventions, and outcome measures, a meta-analysis was not conducted. Findings were organised according to key domains, including study characteristics, types of interoception-based interventions, and reported executive functioning and behavioural outcomes. Patterns across studies were identified and descriptively summarised to highlight similarities, differences, and gaps in the existing literature.

### 2.7. Critical Appraisal

A formal critical appraisal or risk-of-bias assessment was not undertaken. The purpose of this scoping review was to map the range and characteristics of interoception-based interventions and their reported outcomes across heterogeneous study designs rather than to estimate intervention effectiveness or grade the certainty of evidence. Methodological characteristics relevant to interpretation, including study design, sample size, presence of a comparison group, and follow-up, were nevertheless considered descriptively when interpreting the findings.

## 3. Results

### 3.1. Study Selection

The literature search identified 443 records, including 99 from PubMed, 207 from Scopus, and 137 from WoS. After removing 281 duplicate records, 162 records underwent title and abstract screening. Following screening, 147 records were excluded, leaving 15 full-text articles for eligibility assessment. Six studies were excluded due to ineligible intervention (*n* = 1), ineligible age group (*n* = 2), or ineligible study design (*n* = 3). Ultimately, nine studies met the eligibility criteria and were included in this scoping review (Figure 1).

### 3.2. Characteristics of Included Studies

From the nine studies that were included in this scoping review, two were RCTs [10,26], three were one-group pretest–posttest studies [2,5,9], two were quasi-experimental studies [4,19], one mixed-methods quasi-experimental study with waitlist control [27] and one clinical case report [28]. Four studies included comparison-control groups [10,19,26,27].

Across all studies, a total of 391 people participated, ranging from 1 [28] to 125 [10] in each study. Participants ranged in age from 4 [28] to 23 [10] years, with the majority being children and early adolescents (4–14 years). One study [2] included children and young adults (9–19 years) and one study focused on female undergraduate students (17–23 years) [10]. Regarding diagnoses, the study included a heterogeneous population. Diagnoses being reported were autism spectrum disorder [2,4], functional abdominal pain [9], social anxiety disorder [26], learning disabilities [19], avoidant restrictive food intake disorder [28] and mixed diagnoses in special education [5]. One study included healthy preadolescents [27] and one included students with high or low anxiety sensitivity [10].

Overall, the included studies demonstrated considerable heterogeneity in terms of sample size, participants’ age, clinical characteristics, and study design. Nevertheless, they shared an overall focus on investigating interventions targeting interoception or incorporating interoceptive components. Most studies evaluated the feasibility, effectiveness, or impact of these interventions on outcomes related to interoceptive awareness, emotion regulation, executive functioning, behavioural and self-regulation outcomes, pain, anxiety, or stress regulation [2,4,5,9,10,19,26,27]. In contrast, Zucker et al. [28] primarily focused on describing the structure and clinical implementation of the Feeling and Body Investigators (FBI)-ARFID intervention rather than formally evaluating intervention outcomes.

### 3.3. Characteristics of Interoception-Based Interventions (RQ1)

The nine included studies implemented a range of interoception-based intervention approaches, all of which focused on enhancing awareness, identification, and interpretation of internal bodily sensations. Across studies, interoception was primarily targeted through experiential, educational, and exposure-based components designed to strengthen children’s ability to notice, label, and make sense of physiological states such as arousal, pain, hunger, fatigue, and emotional activation [2,4,5,9,10,19,26,27,28].

Regarding intervention content and theoretical approaches, two main intervention models were identified, while the remaining studies employed distinct interventions incorporating interoceptive components. The first model comprised “The Interoception Curriculum”, implemented in three studies, which combined body-focused experiments, structured vocabulary development (descriptor menus), body-check activities, and body mapping to enhance interoceptive awareness and self-regulation [2,4,5]. The second model included acceptance-based interoceptive exposure interventions based on the “FBI” framework, implemented in two studies, which combined psychoeducation, body mapping, guided interoceptive exposure, acceptance-based strategies for reinterpreting bodily sensations, experiential learning, and active caregiver involvement to promote more adaptive interpretations of internal bodily states [9,28]. The remaining studies employed diverse intervention approaches incorporating interoceptive components. Miguez et al. [27] investigated an enactive and playful programme integrating interoceptive and proprioceptive awareness practices, movement-based activities, and reflective group discussions to promote stress regulation and social integration. Sabourin et al. [10] examined an exercise-based interoceptive exposure protocol using repeated running to reduce maladaptive cognitive and affective responses to physiological sensations. Asbrand et al. [26] evaluated a cognitive behavioural therapy programme incorporating psychoeducation, cognitive restructuring, social skills training, and graduated exposure techniques. Finally, Kumar and Swathi [19] investigated an occupational therapy programme integrating body-awareness activities with emotional awareness and academic self-regulation training.

With regard to intervention duration across studies, the included interventions ranged from approximately 7 weeks to 6 months. Brief interventions were reported in three studies [4,5,9], while two studies implemented interventions of approximately 10–12 weeks [10,26]. One study extended over 25 weeks [2], whereas Zucker et al. (2019) delivered a multi-phase programme combining weekly and bi-monthly sessions over an overall period of approximately 3–4 months [28]. The remaining two studies implemented longer-term interventions of approximately 6 months [19,27].

In relation to intervention delivery formats, studies included individual, group, and hybrid approaches. Individual-based delivery was reported in two acceptance-based interoceptive exposure studies [9,28]. Group-based formats represented the most common approach and were used in the majority of studies, including classroom- and group-based interventions [2,4,5,19,26,27]. A hybrid format, combining initial structured sessions with predominantly independent practice, was used in the exercise-based interoceptive exposure study [10].

Intervention delivery providers across the studies reflected a wide range of clinical and research backgrounds. In two studies, interventions were delivered by multidisciplinary clinical teams comprising mental health and medical professionals [9,28]. Occupational therapists played a central role in three studies, either independently or in collaboration with trainees or school staff [2,4,5]. One study was delivered by trained cognitive behavioural therapists and clinical psychologists using a manualised protocol [26], while another occupational therapy-based study reported therapist involvement without clearly specified professional roles [19]. The remaining two studies were implemented by research teams [10,27], occasionally in collaboration with classroom teachers [27].

A summary of study characteristics, participant profiles, intervention approaches, delivery formats, providers, and intervention duration is provided in Table 1.

### 3.4. Executive Functioning Outcomes (RQ2)

Executive functioning outcomes were evaluated in three out of nine included studies. The Behavior Rating Inventory of Executive Function 2 (BRIEF-2) was the main assessment tool, allowing for comparison of outcomes [2,5,19]. Most improvements concerned mainly regulatory domains such as emotional regulation, behavioural regulation and cognitive flexibility. Specifically, improvements were observed in executive functioning and global executive functioning (BRIEF-2 total score) [2,19], emotional control and shift [2,5], working memory, planning and organisation, cognitive and behavioural regulation [2]. None of the three studies reported deterioration in executive functioning outcomes. However, Initiate and Task Monitor did not change significantly in the longest intervention [2].

Academic self-regulation was additionally assessed using the Academic Self Regulation Questionnaire for Learning Disabilities (SRQ-ALD) by Kumar and Swathi [19], where significant improvements were also reported in the experimental group as opposed to the control group.

Across all three studies, improvements were consistently reported in executive functioning following the intervention despite the heterogeneity of interventions and populations.

### 3.5. Behavioural Outcomes (RQ3)

Among the included studies, eight assessed behavioural outcomes [2,4,5,9,10,26,27,28]. Significant heterogeneity was observed in the behavioral outcome measures used, including standardized questionnaires [2,4,5,9,10,26], interviews [9,26,27], behavioral tasks, physiological measures [26,27], and qualitative/clinical observations [28].

Behavioral outcomes encompassed multiple domains rather than a single construct reflecting the diversity of the included interventions and study populations. Outcomes included emotional [2,4,5,9,28], social functioning [27], pain-related [9], eating-related [28], and stress- and anxiety-related behaviors [10,26,27].

Regarding outcomes related to emotional functioning and awareness, Zucker et al. [9] assessed negative effects such as sadness and nervousness and observed a significant reduction, while positive emotions, including calmness, happiness, and cheerfulness, remained unchanged. Additionally, three studies focused on domains related to emotional response, emotional awareness and general emotion and had similarly significant improvements [2,4,5]. Participants in these studies could notice and make meaning or connect body signals with specific emotions [4] and demonstrate greater emotional awareness of bodily needs and states, as well as affective emotion [5]. Finally, while behavioural changes were observed by Kumar and Swathi [19], the study was considered within the executive outcomes category due to the use of the BRIEF-2 which primarily assesses executive functioning.

Social functioning outcomes were reported only in one study [27]. The intervention was associated with improvements in social preference, stress regulation and social integration. Specifically, improvements in interactions with peers and family were mentioned, as well as an increase in their overall social well-being with fewer conflicts and improved communication.

As for pain-related outcomes, in the study of Zucker et al. [9] significant reductions in pain intensity and pain-related distress were reported, reaching up to a 50–70% decrease. Lower but still significant, the category of pain interference decreased approximately 15%.

Eating-related outcomes concerned eating behavior, food intake, and food-energy relationship. The interoception-based intervention helped to reinterpret bodily sensations as informative and manageable rather than confusing or aversive. Furthermore, it led to a more accurate understanding of how food intake affects bodily function and a better estimation of the amount of food required to meet energy needs [28].

Finally, three studies examined stress and anxiety-related outcomes [10,26,27]. Findings were generally positive for anxiety, but less consistent regarding stress indicators and physiological responses. Sabourin et al. [10] reported reduced anxiety sensitivity and more adaptive cognitive and affective responses to exercise. The reduction in anxiety sensitivity was associated with improved ability of the participants to manage their sensations and interpreting them less destructively. One study [27] evaluated chronic stress through hair cortisol concentration and the intervention group showed a significant reduction in chronic stress. In contrast, in the study of Asbrand et al. [26] no improvement was found in objective physiological responses of stress (heart rate and electrodermal activity) after the intervention.

The executive functioning and behavioural outcomes identified across the included studies are summarized in Table 2, including the assessment methods used and the main outcomes reported across interventions.

## 4. Discussion

### 4.1. Summary of Main Findings

The present scoping review examined the available literature on interoception-based interventions and their impact on executive functioning and behavioural outcomes in children and young adults. The included studies implemented a broad range of interoception-based interventions that differed in their theoretical approaches, intervention content, duration, delivery formats, and intervention providers. Regarding executive functioning, the three studies that assessed this outcome consistently reported positive changes, particularly in behavioural regulation, emotional regulation, and academic self-regulation [2,5,19]. Behavioural outcomes were investigated more extensively across multiple domains, with most studies reporting overall positive outcomes. However, the substantial heterogeneity across the included studies limits the ability to draw definitive and generalisable conclusions.

### 4.2. Interpretation of Findings

In contrast to the findings related to behavioural outcomes, evidence regarding executive functioning was more limited, as only three of the included studies examined this outcome. Overall, these studies reported positive trends, particularly in behavioural regulation, emotional regulation, and academic self-regulation, whereas little evidence was identified for improvements in core executive functions such as working memory, inhibitory control, and cognitive flexibility [2,5,19]. This pattern may be explained by the fact that most interoception-based interventions were primarily designed to enhance interoceptive awareness and self-regulation rather than directly target higher-order executive processes. According to developmental models of executive functioning, core executive functions develop progressively throughout childhood and adolescence and generally require targeted and sustained practice to demonstrate measurable improvements [6]. From an interoceptive perspective, increased awareness and interpretation of internal bodily signals may first strengthen self-regulation, which could subsequently support executive functioning through indirect pathways rather than direct cognitive change [3,29,30].

The most consistent findings of the present scoping review were observed in behavioural outcomes, particularly in emotion regulation and self-regulation, with several studies reporting improvements following interoception-based interventions [2,4,5,19]. Positive changes were also reported in behavioural difficulties, while individual studies described improvements in anxiety, eating behaviours, pain perception, and social functioning, suggesting that the potential effects of these interventions may extend across multiple aspects of everyday functioning [2,4,5]. These findings are theoretically plausible, as interoception enables individuals to detect, interpret, and integrate internal bodily signals that contribute to emotional awareness and the regulation of adaptive behavioural responses [31,32]. Greater awareness of internal bodily states may facilitate the recognition of emotional experiences, promote more adaptive responses to environmental demands, and support the implementation of effective self-regulation strategies [3,17,18]. Collectively, these findings suggest that interoception-based interventions may have the potential to support emotional and behavioural functioning in children and young adults and may inform the development of future clinical and educational interventions. Physiological findings related to stress were less consistent and should be interpreted separately from questionnaire-based behavioural outcomes. Miguez et al. [27] reported a reduction in hair cortisol concentration, whereas Asbrand et al. [26] found no change in heart rate or electrodermal activity. These measures capture different aspects and time scales of physiological stress and are therefore not directly comparable. Differences in participant populations, intervention characteristics, and assessment timing may also contribute to the variation in findings; however, the small number of studies prevents these factors from being examined systematically. Given the limited number of studies and the differences in methods, the current evidence does not establish a consistent physiological effect of interoception-based interventions.

Another important finding of the present scoping review was the considerable variation in the characteristics of the included interoception-based interventions. Differences were observed in intervention protocols, programme duration, intervention providers, and participant populations; however, most studies reported overall positive outcomes despite this heterogeneity [2,4,5,19]. This variability is not unexpected, as interoception represents an emerging area of practice within paediatric occupational therapy, and its clinical application continues to evolve across different settings and populations [7]. Therefore, the positive findings observed across the included studies may reflect the importance of shared therapeutic principles, such as enhancing interoceptive awareness, improving the recognition of internal bodily signals, and strengthening emotional awareness, rather than adherence to a specific intervention protocol [18]. This heterogeneity also limits what can be inferred from the apparent similarity of findings across studies. Because the studies differed in population, intervention content, duration, delivery format, and outcome measurement, similar improvements may not reflect the same underlying mechanism and cannot be assumed to generalize across populations or settings. It is also difficult to determine whether reported outcomes were related specifically to the interoceptive components or to other therapeutic elements delivered alongside them. Future studies should describe intervention components, intensity, and duration more consistently and, where possible, use comparable validated outcome measures.

### 4.3. Research Gaps

Despite the encouraging findings, several important gaps remain in the existing literature on interoception-based interventions. First, the available evidence remains limited, with only a small number of studies and just two RCTs published to date. In addition, most studies included relatively small samples, limiting the strength of the available evidence. Executive functioning has also received considerably less attention than emotion regulation and behavioural outcomes, leaving its relationship with interoception insufficiently understood. Furthermore, the use of diverse assessment tools limits comparisons across studies, while the limited availability of follow-up data makes it difficult to determine the long-term sustainability of intervention effects.

### 4.4. Limitations

The limitations of this scoping review included a relatively small number of studies, all of which were published in the English language. The included studies were characterised by relatively small sample sizes, including one case study, while two had a pilot design and one was a feasibility study. Although a formal critical appraisal was not undertaken, the overall evidence base was methodologically uneven. Only two of the nine studies were randomized controlled trials, and four included a comparison or control group. Several studies used one-group pre/post, quasi-experimental, pilot, feasibility, or case-based designs, often with small samples and limited follow-up. These features increase uncertainty around the reported changes and make it difficult to separate intervention effects from maturation, contextual influences, or other concurrent factors. The findings should therefore be interpreted as a mapping of reported outcomes rather than as evidence of intervention efficacy. These methodological characteristics are presented to contextualize the evidence and should not be interpreted as formal study-level quality or risk-of-bias ratings. This limits the methodological strength of the evidence and reduces the generalisability of the findings. Considerable heterogeneity was observed in both the interventions implemented and the populations studied, as participants included both neurotypical and neurodivergent populations. The lack of high-quality experimental designs, particularly RCTs, limits the internal validity of the findings and the ability to establish a direct relationship between the interventions and the observed outcomes. No critical appraisal or risk of bias assessment was undertaken; therefore, the methodological quality of the included studies was not formally evaluated, and the findings should be interpreted with caution.

### 4.5. Implications for Practice and Future Research

The findings of the present review highlight the potential value of interoception-based interventions as a complementary approach for supporting emotion regulation and self-regulation in children and young adults. Their integration may inform clinical and educational practice within occupational therapy, psychology, multidisciplinary teams, school-based interventions, and early intervention programmes and such approaches may contribute to the development of individualized intervention.

Future research should include larger and more representative samples and employ RCTs to strengthen the quality and generalizability of the available evidence [3,7]. In addition, longer-term follow-up assessments are needed to determine whether the effects of interoception-based interventions are maintained over time [2,4]. Greater standardization of both intervention protocols and validated outcome measures is also required [3,7]. Finally, further research should place greater emphasis on examining the effects of interoception-based interventions on executive functioning, because it remains underexplored [2,5,19].

## 5. Conclusions

This scoping review mapped the available scientific evidence on interoception-based interventions in children and young adults by identifying the types of interventions that have been implemented and mapping their effects.

Overall, the findings suggest that these interventions may have a positive effect, primarily on emotion regulation and behavioural outcomes, whereas the available evidence regarding executive functioning remains limited. Despite these encouraging findings, the existing literature is characterised by a relatively small number of studies and methodological heterogeneity in both the intervention protocols and the outcome assessment tools, which limits the methodological strength and the generalisability of the evidence.

Nevertheless, the review highlights the growing clinical and research interest in interoception as a potential intervention approach for promoting self-regulation and executive functioning. By mapping the available evidence and identifying existing knowledge gaps, this review provides a foundation for future research and may contribute to the development of interoception-based interventions with greater methodological consistency. Additional well-designed studies, larger sample sizes, validated assessment tools, and long-term follow-up are required to establish the effectiveness of these interventions and to further clarify their effects.

## Figures and Tables

**Figure 1:**
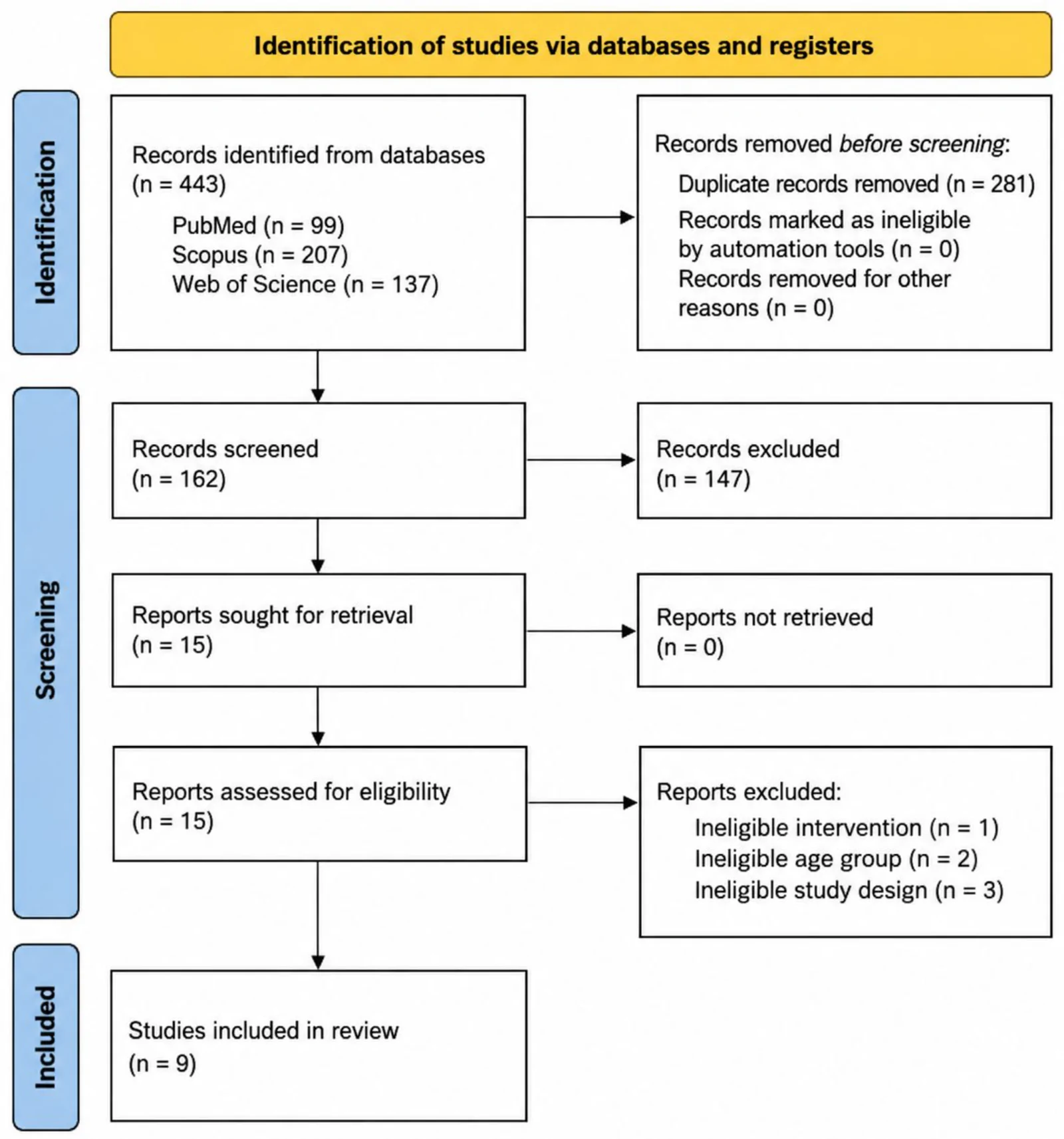
PRISMA flow diagram.

**Table 1:** Characteristics of included studies and interoception-based interventions.

Author(s), Year	Population (N, Age)	Study Design	Intervention Approach	Duration/Format
Zucker et al., 2017 [9]	Children with FAP (*n* = 24; 5–9 years)	Pilot pretest–posttest intervention study	FBI	10 weeks; individual
Mahler et al., 2024 [5]	Students with mixed diagnoses in special education (*n* = 9; 11–14 years)	One-group pretest–posttest study	The Interoception Curriculum	7 weeks; group/classroom-based
Hample et al., 2020 [4]	Children with ASD (*n* = 8; 6–13 years)	Quasi-experimental pretest–posttest study	The Interoception Curriculum	8 weeks; group-based
Asbrand et al., 2020 [26]	Children with SAD (*n* = 119; 9–13 years)	RCT	CBT with interoceptive components	12 weeks; group-based
Kumar & Swathi, 2024 [19]	Children with LD (*n* = 50; 7–11 years)	Quasi-experimental study	Interoceptive occupational therapy programme	6 months; group-based
Zucker et al., 2019 [28]	Child with ARFID (*n* = 1; 4 years)	Clinical case report	FBI–ARFID intervention	3–4 months; individual
Miguez et al., 2026 [27]	Healthy preadolescents (*n* = 41; 10–11 years)	Mixed-methods quasi-experimental study with waitlist control	Enactive and playful interoceptive/proprioceptive programme	6 months; group-based
Mahler et al., 2022 [2]	Children and young adults with ASD (*n* = 14; 9–19 years)	One-group pretest–posttest study	The Interoception Curriculum	25 weeks; classroom-based
Sabourin et al., 2015 [10]	Female undergraduate students with high/low anxiety sensitivity (*n* = 125; 17–23 years)	RCT	CBT-based interoceptive exposure through exercise	10 weeks; hybrid

ASD = Autism Spectrum Disorder; ARFID = Avoidant Restrictive Food Intake Disorder; CBT = Cognitive Behavioural Therapy; FBI = Feeling and Body Investigators; FAP = Functional Abdominal Pain; LD = Learning Disabilities; RCT = Randomized Controlled Trial; SAD = Social Anxiety Disorder.

**Table 2:** Summary of outcome domains, assessment methods, reported outcome patterns, and main findings across the included studies.

Author(s), Year	Outcome Domain	Assessment Tool/Method	Main Findings	Reported Outcome Pattern	Comparison/Statistical Context
Kumar & Swathi, 2024 [19]	Executive functioning; self-regulation	BRIEF-2; SRQ-ALD	Improvements in executive functioning and academic self-regulation compared with the control group.	Improvement reported	Experimental group improved compared with the control group; significant improvement reported in academic self-regulation.
Mahler et al., 2022 [2]	Executive functioning; behavioural outcomes	BRIEF-2; questionnaires	Improvements in executive domains and emotional/behavioural regulation; no significant changes in Initiate and Task Monitor.	Mostly improved; no significant change in Initiate and Task Monitor	One-group pre/post design; no control group. Initiate and Task Monitor did not change significantly.
Mahler et al., 2024 [5]	Executive functioning; behavioural outcomes	BRIEF-2; standardized measures	Improvements in emotional control, cognitive flexibility, and emotional awareness.	Improvement reported	One-group pre/post design; no control group; significant improvements reported in emotional outcomes.
Asbrand et al., 2020 [26]	Behavioural outcomes	Questionnaires; physiological measures	Improvements in anxiety-related outcomes; no changes in physiological stress indicators.	Mixed: anxiety-related improvement; no physiological change	RCT with comparison group; anxiety-related improvements were reported, but no improvement was found in heart rate or electrodermal activity.
Hample et al., 2020 [4]	Behavioural outcomes	Standardized measures	Improved emotional awareness and recognition of emotional states.	Improvement reported	Quasi-experimental pre/post design; no control group; significant improvements in emotional awareness were reported.
Miguez et al., 2026 [27]	Behavioural outcomes	Interviews; physiological measures	Improvements in social functioning and reduction in chronic stress.	Improvement reported	Quasi-experimental study with waitlist control; significant reduction in hair cortisol was reported in the intervention group.
Sabourin et al., 2015 [10]	Behavioural outcomes	Standardized questionnaires	Reduced anxiety sensitivity and improved regulation of bodily sensations.	Improvement reported	RCT with comparison group; reductions in anxiety sensitivity and more adaptive responses to bodily sensations were reported.
Zucker et al., 2017 [9]	Behavioural outcomes	Questionnaires; interviews	Reductions in negative emotions, pain intensity, pain-related distress, and pain interference.	Improvement reported	One-group pre/post design; no control group; significant reductions in negative affect, pain intensity, pain-related distress, and pain interference were reported.
Zucker et al., 2019 [28]	Behavioural outcomes	Qualitative/clinical observations	Improved interpretation of bodily signals related to eating and food-energy needs.	Descriptive clinical improvement	Clinical case report; descriptive clinical findings only, with no comparison group or inferential analysis.

BRIEF-2 = Behavior Rating Inventory of Executive Function–Second Edition; SRQ-ALD = Academic Self-Regulation Questionnaire for Learning Disabilities.

## Data Availability

No new data were created or analyzed in this study. Data sharing is not applicable to this article.

## Abbreviations

The following abbreviations are used in this manuscript:

**Abbreviation**	**Full term**
ARFID	Avoidant/restrictive food intake disorder
ASD	Autism spectrum disorder
BRIEF-2	Behavior Rating Inventory of Executive Function, Second Edition
CBT	Cognitive behavioral therapy
DOI	Digital object identifier
FAP	Functional abdominal pain
FBI	Feeling and Body Investigators
FBI–ARFID	Feeling and Body Investigators—ARFID division
LD	Learning disabilities
MeSH	Medical Subject Headings
OSF	Open Science Framework
PRISMA	Preferred Reporting Items for Systematic Reviews and Meta-Analyses
PRISMA-ScR	Preferred Reporting Items for Systematic Reviews and Meta-Analyses extension for Scoping Reviews
R&D	Research and development
RCT	Randomized controlled trial
RQ	Research question
SRQ-ALD	Academic Self-Regulation Questionnaire for Learning Disabilities
tiab	Title/abstract search field in PubMed
TITLE-ABS-KEY	Title, abstract, and keywords search field in Scopus
TS	Topic search field in Web of Science
WoS	Web of Science

## Appendix A

**Table A1:** Search Strings Used for Literature Retrieval.

Database	Search Strings
PubMed	(“Interoception”[Mesh] OR interoception[tiab] OR interoceptive[tiab] OR “interoceptive awareness”[tiab] OR “body awareness”[tiab]) AND (“Child”[Mesh] OR “Adolescent”[Mesh] OR child*[tiab] OR adolescent*[tiab]) AND (intervention*[tiab] OR therap*[tiab] OR training[tiab]) AND (“executive function*”[tiab] OR “working memory”[tiab] OR inhibition[tiab] OR “cognitive flexibility”[tiab] OR “self-regulation”[tiab] OR behavior*[tiab] OR “emotional regulation”[tiab] OR externalizing[tiab] OR internalizing[tiab])
Scopus	TITLE-ABS-KEY( (interoception OR interoceptive OR “interoceptive awareness” OR “body awareness”) AND (child* OR adolescent*) AND (intervention* OR therap* OR training) AND (“executive function*” OR “working memory” OR inhibition OR “self-regulation” OR behavior* OR “emotional regulation”) )
WoS	TS=( (interoception OR interoceptive OR “interoceptive awareness” OR “body awareness”) AND (child* OR adolescent*) AND (intervention* OR therap* OR training) AND (“executive function*” OR “working memory” OR inhibition OR “self-regulation” OR behavior* OR “emotional regulation”) )
